# The Cytotoxic Effect of Septic Plasma on Healthy RBCs: Is Eryptosis a New Mechanism for Sepsis?

**DOI:** 10.3390/ijms241814176

**Published:** 2023-09-16

**Authors:** Matteo Marcello, Grazia Maria Virzì, Davide Marturano, Massimo de Cal, Nicola Marchionna, Luca Sgarabotto, Silvia De Rosa, Claudio Ronco, Monica Zanella

**Affiliations:** 1 Department of Nephrology, Dialysis and Transplant, St Bortolo Hospital, 36100 Vicenza, Italymonica.zanella@aulss8.veneto.it (M.Z.); 2 IRRIV-International Renal Research Institute, 36100 Vicenza, Italy; 3 Nephrology, Dialysis and Transplantation Unit, Department of Medicine, University of Padova, 35100 Padova, Italy; 4 Centre for Medical Sciences-CISMed, University of Trento, Via S. Maria Maddalena 1, 38122 Trento, Italy; 5 Anesthesia and Intensive Care, Santa Chiara Regional Hospital, APSS, 38122 Trento, Italy

**Keywords:** sepsis, eryptosis, endotoxin, EAA

## Abstract

Sepsis is a life-threatening multiple-organ dysfunction induced by infection and is one of the leading causes of mortality and critical illness worldwide. The pathogenesis of sepsis involves the alteration of several biochemical pathways such as immune response, coagulation, dysfunction of endothelium and tissue damage through cellular death and/or apoptosis. Recently, in vitro and in vivo studies reported changes in the morphology and in the shape of human red blood cells (RBCs) causing erythrocyte death (eryptosis) during sepsis. Characteristics of eryptosis include cell shrinkage, membrane blebbing, and surface exposure to phosphatidylserine (PS), which attract macrophages. The aim of this study was to evaluate the in vitro induction of eryptosis on healthy RBCs exposed to septic plasma at different time points. Furthermore, we preliminary investigated the in vivo levels of eryptosis in septic patients and its relationship with Endotoxin Activity Assay (EAA), mortality and other biological markers of inflammation and oxidative stress. We enrolled 16 septic patients and 16 healthy subjects (no systemic inflammation in the last 3 months) as a control group. At diagnosis, we measured Interleukin-6 (IL-6) and Myeloperoxidase (MPO). For in vitro study, healthy RBCs were exposed to the plasma of septic patients and CTR for 15 min, 1, 2, 4 and 24 h. Morphological markers of death and eryptosis were evaluated by flow cytometric analyses. The cytotoxic effect of septic plasma on RBCs was studied in vitro at 15 min, 1, 2, 4 and 24 h. Healthy RBCs incubated with plasma from septic patients went through significant morphological changes and eryptosis compared to those exposed to plasma from the control group at all time points (all, *p* < 0.001). IL-6 and MPO levels were significantly higher in septic patients than in controls (both, *p* < 0.001). The percentage of AnnexinV-binding RBCs was significantly higher in septic patients with EAA level ≥0.60 (positive EAA: 32.4%, IQR 27.6–36.2) compared to septic patients with EAA level <0.60 (negative EAA: 14.7%, IQR 5.7–30.7) (*p* = 0.04). Significant correlations were observed between eryptosis and EAA levels (Spearman rho2 = 0.50, *p* < 0.05), IL-6 (Spearman rho2 = 0.61, *p* < 0.05) and MPO (Spearman rho2 = 0.70, *p* < 0.05). In conclusion, we observed a quick and great cytotoxic effect of septic plasma on healthy RBCs and a strong correlation with other biomarkers of severity of sepsis. Based on these results, we confirmed the pathological role of eryptosis in sepsis and we hypothesized its use as a biomarker of sepsis, potentially helping physicians to face important treatment decisions.

## 1. Introduction

Sepsis is described as a life-threatening multi-organ dysfunction originated by a dysregulated host response to infection, induced by different alterations in numerous biochemical pathways [1]. Despite advancements in diagnostic and therapeutic tools, the declared incidence of sepsis is increasing and it accounts for one of the principal causes of mortality and critical illness worldwide [2]. The pathogenesis of sepsis is very complex, and it involves several mechanisms, such as infection, inflammation, oxidative stress, immune system dysregulation, blood coagulation, endothelium dysfunction, tissue damage, elevated cellular death and/or apoptosis [3].

Gram-negative infection is one of the many causes of sepsis and it is associated with endotoxemia-induced alterations in the immune system [4]. Recent advances in the diagnostic tool of sepsis allow detection of endotoxin in whole blood using neutrophil-dependent chemiluminescence [5]. This has important diagnostic and therapeutic relevance given the possibility of removing endotoxins from the bloodstream with Extracorporeal Blood Purification Therapy. IL-6 and MPO are involved in the altered inflammatory response and they are commonly used as biomarkers for diagnosis and evaluation of severity in sepsis [6,7]. IL-6 is a pleiotropic multifunctional cytokine that acts as endogenous pyrogen and one of the main stimuli for the secretion of acute-phase proteins from the liver [8,9]. Therefore, increased levels of IL-6 do not necessarily reflect a pathological role in sepsis. Myeloperoxidase (MPO) is an enzyme stored in the neutrophil granules that act as a component of innate immunity, participating in the phagocytosis of bacteria [10].

During sepsis, an alteration between pro-inflammatory and anti-inflammatory cytokines exists with the activation of inflammasomes and expression of iron-associated [11]. Inflammation is therefore highly correlated with sepsis-induced anaemia.

Recently, in vitro and in vivo studies reported changes in the morphology and in the shape of human red blood cells (RBCs) causing erythrocyte death (eryptosis) during sepsis. Erythrocyte RBCs have a greatly peculiar and well-organized membrane composition and structure, which react to xenobiotic and endogenous compounds, such as inflammatory molecules, cytokines, toxins and oxidative stress molecules [12,13,14,15]. Nonetheless, these cells are very susceptible to damage and are characterized by a specific type of programmed cell death, comparable to apoptosis, defined as eryptosis [12,13,14]. In particular, the biological mechanism of eryptosis is very intricated and it is defined by different steps, such as cell shrinkage, membrane blebbing, and surface exposure on the outer membrane of phosphatidylserine (PS) that attract macrophages. PS-exposing RBCs were degraded and cleaned out from circulation [16,17,18,19]. During eryptosis, RBCs induce CD4+/CD8+ T-cell proliferation through the release of exosomes. The subsequent release of haemoglobin from damaged RBCs induces activation of macrophage, phagocytosis and expression of heme oxygenase 1 leading to inhibition of oxidative damage [20].

Eryptosis is known to be involved in the pathogenesis of many clinical conditions, such as anaemia, diabetes, uremia, fever, peritonitis and dehydration [16,17,21,22,23,24,25,26,27]. During sepsis, eryptosis is associated with a higher severity of disease and mortality rate [28]. Furthermore, compared with other biomarker of sepsis, eryptosis is easy to assess and less expensive [29]. Nonetheless, very little is known about the exact mechanism of eryptosis and its timing in the pathogenesis of sepsis.

The aim of this study was to evaluate the in vitro induction of eryptosis on healthy RBCs exposed to septic plasma at different time points. Furthermore, we preliminary investigated the in vivo levels of eryptosis in septic patients and its relationship with Endotoxin Activity Assay (EAA), mortality and other biological markers of inflammation and oxidative stress.

## 2. Results

### 2.1. Subjects Baseline Characteristics

A total of 16 septic patients were enrolled in this pilot study. The mean age was 58.25 ± 14.4 years and 69% of these patients were males. In the case group, seven (43.8%) patients had diabetes, five (31.3%) had hypertension and five (31.3%) had cardiovascular disease (CVD). Four patients had Chronic Kidney Disease (CKD), one patient was treated by peritoneal dialysis and one patient received kidney transplantation. Table 1 reported baseline characteristics for all patients (Table 1).

In the control group, the mean age of 17 healthy volunteers was 51.4 ± 8.2 years and 53% of these subjects were male. The biochemical parameters of all subjects were within the range. Diabetes, CVD and CKD were not found in this group.

### 2.2. EAA Evaluation and Outcome

We evaluated EAA in all patients. The median value of EAA was 0.55 (IQR 0.42–0.77) in our septic population. In particular, four patients had an EAA ≤0.39 (low level) and the median EAA was 0.3 (IQR 0.2–0.37) in this group. Six patients had an EAA value ≥0.60 (high level). In this group, the median value of EAA was 0.28 (IQR 0.76–0.88). Six patients had an EAA value in the intermediate range (median: 0.48, IQR 0.51–0.54).

Six (37.5%) patients died during the ICU stay. The 6-month mortality was 20% (two patients).

### 2.3. In Vitro Exposure to Septic Plasma and Induction of Eryptosis

**Figure 1:**
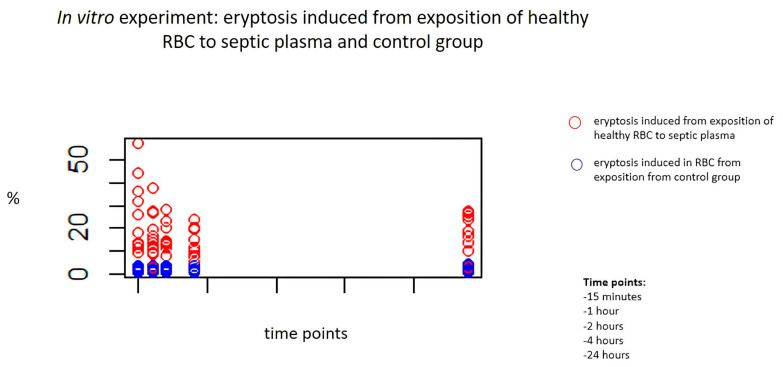
Induced eryptosis in our in vitro experiment. Blu dots indicate degree of eryptosis induced in RBC from exposition to plasma from control group, red dots indicate eryptosis induced from exposition of healthy RBC to septic plasma. Healthy RBCs incubated with plasma from septic patients demonstrated a significant increase in eryptosis compared to healthy RBCs exposed to plasma from control group at all time points (all, *p* < 0.001).

The cytotoxic effect of septic plasma on RBCs was studied in vitro at 15 min, and then after 1, 2, 4 and 24 h. Healthy RBCs incubated with plasma from septic patients demonstrated a significant derangement of cell morphology and a significant increase in eryptosis compared to healthy RBCs exposed to plasma from the control group at all time points (all, *p* < 0.001). Table 2 reports the level of eryptosis on RBCs treated with septic and healthy plasma (Table 2). Figure 1 shows induced eryptosis in our in vitro experiment (red: case group; blue: control group) (Figure 1).

**Figure 2:**
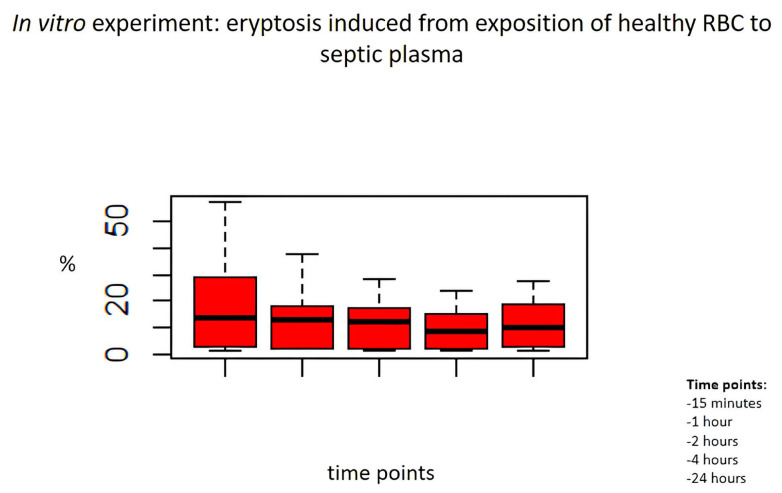
In vitro experiment: eryptosis induced from exposition of healthy RBC to septic plasma. Induced eryptosis by septic plasma was significantly different: at 15 min compared to 1 h (*p* = 0.001), at 1 h compared to 2 h (*p* = 0.02) and at 2 h compared to 4 h (*p* = 0.03). Percentage of eryptosis was similar at 4 and 24 h (*p* = 0.18).

In particular, we analysed the cytotoxic effects of septic plasma at each time point. Figure 2 shows the cytotoxic effects of septic plasma on healthy RBCs (Figure 2). Induced eryptosis by septic plasma showed significantly different results: at 15 min compared to 1 h (*p* = 0.001), at 1 h compared to 2 h (*p* = 0.02) and at 2 h compared to 4 h (*p* = 0.03). The percentage of eryptosis was similar at 4 and 24 h (*p* = 0.18).

### 2.4. In Vivo Eryptosis Evaluation

Eryptosis is characterized by cell shrinkage, cell membrane scrambling and PS exposure at the RBC surface. In order to investigate cell membrane scrambling, cell shrinkage and PS exposure at the RBC surface, markers of eryptosis, and eryptototic RBCs were identified by FS (cell volume dimension) and AnnexinV-binding using flow cytometric analyses. RBCs of septic patients were dramatically deranged in their morphology and the average FS, reflecting cell volume, was significantly higher in case group respect controls (*p* < 0.001).

**Figure 3:**
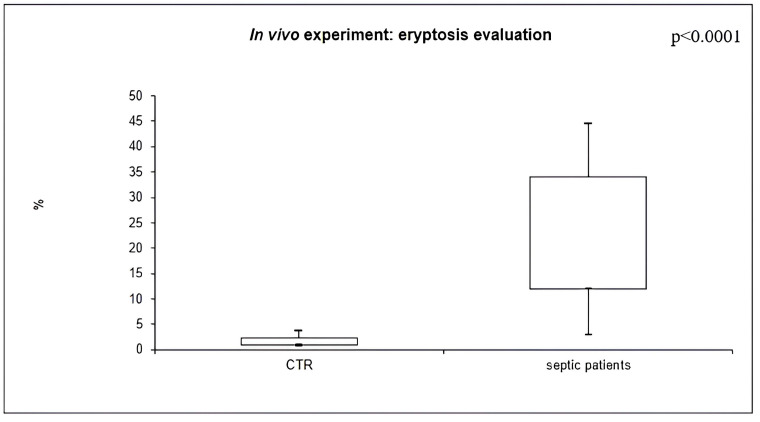
In vivo experiment: eryptosis in septic patients and CTR.

The percentage of AnnexinV-binding RBCs was significantly higher in septic patients (22.1%, IQR 12.2–32.1), compared to healthy subjects (1.4%, IQR 1.0–2.3) (*p* < 0.0001) (Figure 3).

**Figure 4:**
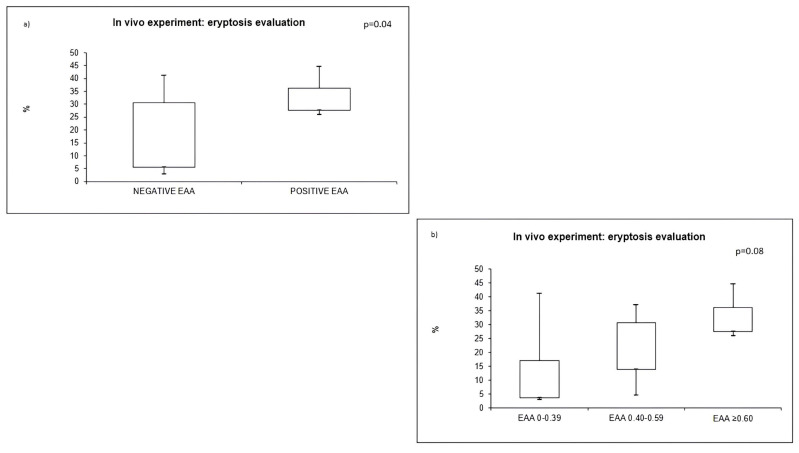
In vivo experiment: eryptosis evaluation and EAA. (**a**) eryptosis in positive and negative EAA, (**b**) eryptosis in low, intermediate and high level of EAA.

Furthermore, we performed a sub-analysis in septic patients: the median percentage of eryptosis did not differ in patients with or without diabetes (*p* = 0.7), with or without hypertension (*p* = 0.9), and with or without CVD (*p* = 0.7). The percentage of AnnexinV-binding RBCs was significantly higher in septic patients with EAA level ≥0.60 (positive EAA: 32.4%, IQR 27.6–36.2), compared to septic patients with EAA level <0.60 (negative EAA: 14.7%, IQR 5.7–30.7) (*p* = 0.04) (Figure 4a). Furthermore, we classified patients into three groups based on EAA value: low level, intermediate level and high level. There was no significant difference in terms of eryptosis among these three groups (*p* = 0.08). However, the *p*-value did not reach statistical significance; there was a tendency towards a lower level of eryptosis in septic patients with a low level of EAA (Figure 4b).

The median percentage of eryptosis did not differ in patients with positive or negative outcomes (15.7%, 4.5–33.9 versus 12.9%, 5.2–22.9; *p* = 0.43).

### 2.5. Relation to Other Biomarkers

We evaluated inflammation (IL-6) and oxidative stress (MPO) biomarkers in our septic population and in healthy subjects. Table 3 reported IL-6 and MPO levels in septic patients and in controls (Table 3). IL-6 and MPO levels were significantly higher in septic patients than in controls (both, *p* < 0.005). The median values of IL-6 and MPO did not differ in patients with EAA levels≥ or <0.60 (*p* = 0.41 and *p* = 0.59, respectively). The median values of IL-6 and MPO did not differ in patients with positive or negative outcomes (*p* = 0.97 and *p* = 0.85, respectively).

Moreover, statistically significant correlations were observed between eryptosis and IL-6 (Spearman rho^2^ = 0.61, *p* < 0.05), MPO (Spearman rho^2^ = 0.70, *p* < 0.05), and EAA levels (Spearman rho^2^ = 0.50, *p* < 0.05).

## 3. Discussion

In this case–control study, we performed both in vitro and in vivo assessments of eryptosis in sepsis. In the in vitro experiment, healthy RBCs were incubated with plasma taken from both septic patients and healthy volunteers. Flow cytometric analyses were performed in order to detect eryptosis. We found a significant alteration in the morphology of cells and an increased degree of eryptosis in RBCs incubated with septic plasma. In particular, septic-induced eryptosis reached its maximum value at 15 min The present experiment suggests that eryptosis occurs very rapidly in the pathogenesis of sepsis. The timing of this phenomenon has to be validated and confirmed by in vivo settings with multiple biological samples at specific time points.

Flow cytometric analyses conducted in both groups confirmed the presence of a dramatic alteration in the morphology of RBCs of septic patients and showed a higher percentage of AnnexinV-binding RBCs compared to healthy subjects. Furthermore, we performed evaluation of EAA, IL-6 and MPO in all patients. Finally, we found a significant correlation between eryptosis and other biomarkers of sepsis such as EAA, IL-6 and MPO. However, the degree of eryptosis did not correlate with comorbidity and mortality.

Alteration of RBCs’ shape and morphology during sepsis has been already described during in vitro studies and subsequently confirmed in humans [30,31].

Even if this phenomenon is well known, the molecular pathway behind it remains to be clarified. An important role is played directly by the pathogen that invades the cytosolic space of RBC and induces a hyperosmolar state, oxidative stress and intracellular Ca^2+^ activity [32]. However, RBCs’ exposure to septic plasma may induce phosphatidylserine translocation and activation of sphingomyelinase leading to eryptosis [29]. In our study, we found similar alterations in the morphology of erythrocytes in both in vitro and in vivo studies. This alteration in morphology occurs also in aged erythrocytes, making them susceptible to phagocytosis by macrophage. RBCs’ destruction depends on the net positive and negative signals sent to macrophages. CD47, a 50 kDa plasma membrane protein, protect healthy RBCs from phagocytosis by sending negative signals to macrophages through specific receptors [33]. However, during sepsis, an increase in cell-free haemoglobin due to hemolysis has been described. The aetiology is multifactorial and associated with hemolytic pathogens, disseminated intravascular coagulation, RBC apoptosis and LPS invasion of erythrocytes [34]. Furthermore, during the acute phase of sepsis, there is secretion of different pro-inflammatory cytokines. IL-10, in particular, can inhibit phagocytosis and represents a biomarker of the immunosuppressive phase of sepsis [35].

In animal model with sepsis induced by LPS, sepsis-free heme worsened the septic state [36]. Endotoxin (LPS) is a component of the cell wall of Gram-negative bacteria and its presence in the blood usually results from bacterial infection or traslocation from gastrointestinal tract. The Endotoxin Activity Assay™ is a chemiluminescent assay and provides an easy and quick tool to determine patient’s endotoxin level. An elevated level of EAA is a significant risk factor to developing severe sepsis and is associated with increased organ dysfunction and mortality [37]. Based on these observations, several therapies targeting endotoxin were studied, in particular in the setting of extracorporeal blood purification therapies. In the EUPHRATES trial, the use of polymyxin-B cartridge in patients with EAA levels between 0.6 and 0.89 was associated with significantly reduced 28-day mortality, adjusted for the APACHE II score and baseline MAP [38]. In this study, we found a strong relationship between eryptosis and EAA level, with a percentage of AnnexinV-binding RBCs significantly higher in septic patients with EAA levels ≥ 0.60. In our analyses, we did not find high percentages of AnnexinV-binding RBCS in patients with intermediate levels of EAA (0.40–0.60), even if a positive trend was found. This could be due to the low number of patients analysed, and further evaluation is needed in this group of patients to validate and confirm these interesting results. Evidence suggests that LPS induce apoptosis by activating cytokine production and release via the secretion of TNF-α [39]. Among these cytokines, IL-6 is able to prolong STAT1 (mediator of the signalling of interferon) activation, induce the expression of major histocompatibility complex (MHC) class I and finally lead directly to apoptosis [40]. This finding could provide a rationale for the strong correlation between eryptosis and EAA and IL-6 levels that results from our analyses. Moreover, in our study, we found a correlation between eryptosis and MPO levels. In a recent observational study, MPO levels measured in critically ill patients at the time of ICU hospitalization allowed us to distinguish septic patients from patients without sepsis. Furthermore, the maximum MPO level measured in the first 48 h was correlated to short-term mortality [41]. MPO is a hemeprotein that synthesized different reactive oxidants necessary for the lysis of the ingested bacteria. Given its role in phagocytosis, we can consider it an executor of the innate immunity. On the other end, eryptosis is an effect of phagocytosis of RBCs induced by the septic milieu. Because innate immunity is the first to take place during infection, the presence of these epiphenomena allows clinicians to assess the severity of sepsis in the very early phase.

Unfortunately, we did not find correlations between eryptosis and comorbidity as we expected. There is evidence that cytokines play an important role in altering the ultrastructure of RBCs, platelets and endothelial cells leading to the development of conditions like atherosclerosis and metabolic syndrome [42].

It is well described that pathogens during the invasion of the bloodstream and organs, induce secretion of pro-inflammatory cytokines such as IL-6 and IL-8 and also cause phagocytes to produce reactive oxygen species. However, these effectors of innate immunity can also affect RBCs and lead to eryptosis. Subsequently, cell-free haemoglobin and free iron are responsible for further damage leading to inflammation, bacterial growth complement activation and disseminated intravascular coagulation [20]. For this reason, we expected to see a strong correlation between the degree of eryptosis and short-term mortality. Probably, the small cohort of patients studied in this pilot study is responsible for this result. Probably, it is necessary to increase the sample size of our cohort to investigate this aspect. Alternative per metterla in positive, eventualmente modifica.

Although the small cohort of patients enrolled, our study provides new evidence on the genesis and pathophysiology of eryptosis in sepsis suggesting a prominent role of LPS in inducing eryptosis and hemolysis. Our preliminary results can be considered hypothesis-generating, and stimulate further explorations. Our in vitro study shows that this phenomenon reaches its peak at the very early phase of sepsis (15 min from incubation of RBCs with septic plasma), pointing out once again the clinical relevance of early diagnosis and prompt therapy. In a prospective study, Reggiori et al. demonstrated that after 3 days of hospitalization, patients with sepsis exhibited lower haemoglobin and hematocrit values compared to patients without sepsis [31]. Our in vitro results suggest that mechanisms leading to the alteration of RBCs and haemoglobin occur in the very first phase of disease and this could help to early identify patients with more severe disease. Even if our in vivo study did not find differences in the degree of eryptosis among patients with positive or negative outcomes, eryptosis is known to be a marker of the severity of the disease. In a retrospective study, Sadaka et al. demonstrated a strong correlation between increased red cell distribution width and mortality among patients with sepsis or septic shock [43]. Furthermore, the strong correlation with EAA level suggests an important role of LPS in inducing apoptosis, clarifying some of the molecular basis of this phenomenon. The positive trend seen in the intermediate EAA group, if confirmed in future studies with a greater number of patients, could represent a further tool in the decision to start Extracorporeal Blood Purification Therapy in patients who do not meet the criteria.

The in vitro assessment of eryptosis at different time points allowed us to define this phenomenon as an early complication in sepsis. Our in vivo study shows an important correlation between this phenomenon and clinical outcome and other biomarkers of sepsis, clarifying important aspects in the aetiology of eryptosis and its potential role as a marker of the severity of disease. We acknowledge some limitations; our study population was too small, and in our in vivo study, we did not evaluate the timing of eryptosis and therefore we could not completely confirm the in vitro data.

To our knowledge, this is the first study that assesses the timing of onset of eryptosis in the pathogenesis of sepsis. Even if the assessment of the timing of eryptosis was conducted only in vitro, we think our experiment represents a further step in the comprehension of eryptosis and a first attempt to integrate this physiopathological epiphenomenon into the clinical setting. This is of particular interest in both diagnostic and therapeutic fields; even if the presence of eryptosis is considered to be a marker of the severity of sepsis, the possibility of detecting its presence in the very early phase of the disease could improve therapeutic management. Another important finding in our study was the correlation between eryptosis and other biomarkers of sepsis. Correlation with IL-6 and MPO could be used in the clinical setting to develop an early severity score. In particular, eryptosis represents an economic test, easy to perform without advanced laboratory equipment. As is known, a therapeutic option in patients with severe sepsis from Gram-negative bacteria is Extracorporeal Blood Purification Therapy with polymyxin B cartridge when EAA is above 0.6. Given the strong relationship with EAA, eryptosis could help to define the severity of disease in those patients in which clinical and laboratory setting does not allow to reach criteria for extracorporeal blood purification therapy, moving one step toward precision medicine. These preliminary findings provide a further rationale for ongoing clinical studies and give an interesting clinical perspective on eryptosis: our results need to be validated in vivo in randomized clinical trials with larger sample sizes.

## 4. Materials and Methods

### 4.1. Subjects

In this case–control study, we enrolled 16 septic patients (“case” group) admitted to the Intensive Care Unit (ICU) of San Bortolo Hospital in Vicenza and 16 healthy subjects (no systemic inflammation in the last 3 months), as a control group, from the Department of Transfusional Medicine of San Bortolo Hospital in Vicenza. Sepsis was defined as a life-threatening organ failure caused by the host’s inappropriate response to infection according to the Sepsis-3 criteria. Organ failure and severity of disease were defined according to the SOFA score [44]. For each patient, we obtained demographic and clinical information at the time of hospitalization including routine hospitalization laboratory analyses. All parameters were reported in a specific data collection form and in a specific dataset. Negative outcome was defined as death during the ICU stay.

### 4.2. Sample Collection

Peripheral venous EDTA blood samples were collected for each patient within 4 h from the diagnosis of sepsis. Samples were processed within 30 min after collection and centrifuged for 10 min at 1600× *g*, to obtain plasma that was immediately separated and stored at –80 °C until use.

### 4.3. Endotoxin Activity Assay (EAA)

Plasma endotoxin activity was determined by the EAA (Endotoxin Activity Assay) (Estor Spa, Milan, Italy). Endotoxin activity levels are expressed as units on a scale ranging from 0 to 1. This diagnostic test is based on a monoclonal antibody that recognizes endotoxin. In particular, LPS activity is calculated on the corresponding oxidative burst of primed neutrophils (complexes of an anti-endotoxin antibody and endotoxin) and is detected via the chemiluminescence methodology [45]. We defined three categories of patients based on their EAA level: low was used for EAA levels between 0.00 and 0.39, high was used for values above 0.60 and finally intermediate for EAA levels between 0.40 and 0.59. According to the literature, values above 0.60 are considered associated with worse outcomes and in these cases, Extracorporeal Blood Purification Therapies may be beneficial [46].

### 4.4. Enzyme-Linked Immunosorbent Assay (ELISA) for IL-6 and MPO

Quantitative determination of plasma Interleukin-6 (IL-6) was executed by Human IL-6 Simple step ELISA Kit-ab178013 (Discovery Drive, Cambridge Biomedical Campus, Cambridge, CB2 0AX, UK) according to manufacturer’s instructions. The concentration values for these molecules were calculated by the extrapolation with standard curves (values of standard tested: 1000-500-250-125-62-5-31.3-15.6-0 pg/mL). All tests were carried out in duplicate.

Quantitative determination of plasma MPO concentration was analysed by Human Instant ELISA kit-BMS2038INST (Invitrogen, Bender MedSystems GmbH, Campus Vienna Biocenter 2, 1030 Vienna, Austria). Preliminary plasma dilution 1:100 was performed for each sample with specific Sample Diluent. MPO determination was performed according to the manufacturer’s protocol and instructions. The levels of this molecule were estimated from the interpolation with the standard curve (values of standard tested 10000-5000-2500-1250-625-313-156-0 pg/mL) based on the manufacturer’s protocol. All tests were executed in duplicate.

Optical density for both testes was read by VICTORX4 Multilabel Plate Reader (PerkinElmer Life Sciences, Waltham, MA, USA) at 450 nm for both molecules.

### 4.5. In Vitro Exposure to Septic Plasma and Induction of Eryptosis

We performed an in vitro study with healthy RBCs exposed to the plasma of septic patients and CTR at different time points. We evaluated the induction of eryptosis during exposure of RBCs to septic plasma in comparison to the exposure to healthy subject plasma.

For this in vitro experiment, 1.5 µL of RBCs from a healthy subject were plated per well in 48-well plates in 300 µL of RPMI supplemented with 2 mM L-glutamine, 100 IU/mL penicillin and 100 µg/mL streptomycin (all from Sigma Chemical Co., St. Louis, MO, USA). Each well was incubated with 10% EDTA plasma from septic patients or controls. We used untreated RBCs as a negative control (RPMI supplemented with 2 mM L-glutamine, 100 IU/mL penicillin and 100 µg/mL streptomycin and 10% of heat-inactivated Fetal Bovine (Serum-Sigma Chemical Co., St. Louis, MO, USA). All RBCs were incubated in standard conditions (37 °C in 5% CO^2^). Each incubation was performed at different times (15 min, 1, 2, 4 and 24 h) in duplicate. Each well was tested twice.

### 4.6. In Vivo Eryptosis

In vivo eryptosis was evaluated in controls and in septic patients on the first day of sepsis. In total, 1μL of leukocyte-depleted RBCs was diluted in 400 μL Ringer solution containing 5 mM CaCl_2_. Then, this solution was diluted 1:1 in Ringer solution containing 5 mM CaCl_2_. 

In total, 1μL of AnnexinV-FITC-conjugated (Beckman Coulter, Brea, CA, USA) was used for eryptosis evaluation (incubation for 20 min under stained protection from light). Finally, 400 μL of Ringer was added to each tube.

### 4.7. Flow Cytometry Evaluation

All eryptosis measurements were taken in freshly isolated RBCs. The analysis was executed by Navios Flow Cytometer (Beckman Coulter, Brea, CA, USA) and 1 μL of AnnexinV–FITC-conjugated (Beckman Coulter, Brea, CA, USA) to identify the subpopulations of the eryptotic RBCs. Cell volume was determined utilizing Forward Scatter (FS). PS avidly binds AnnexinV, which was employed to identify eryptotic cells. Thus, PS exposure at the RBC surface was estimated from FITC–AnnexinV binding. AnnexinV fluorescence intensity was measured with an excitation wavelength of 488 nm and an emission wavelength of 530 nm. RBCs were gated and enumerated by identifying those cells that exposed PS at the RBC surface. A minimum of 100,000 events were collected on each sample.

### 4.8. Statistical Analysis

Statistical analysis was performed using the SPSS Software package(VERSION 11). A *p*-value of <0.05 was considered statistically significant. Graphs were achieved by SPSS and Excel. Categorical variables were expressed as percentages; continuous variables were expressed as mean ± standard deviation (parametric variables) or median and interquartile range (IQR) (nonparametric variables). The Mann–Whitney *U* test or *t* test was used for comparison of two groups when appropriate. The Kruskal–Wallis test or ANOVA test for multiple comparisons was applied to compare the groups when appropriate. Spearman’s rho correlations were calculated to verify the correlation between variables.

## Figures and Tables

**Table 1:** Baseline characteristics.

	Septic Patients, n = 16
Age, years	58.25 ± 14.4
Sex	11 male
Diabetes	7
Hypertension	5
CVD	5
CKD	4
EAA level	0.55, IQR 0.42–0.77

**Table 2:** In vitro induced eryptosis at different time points.

In Vitro Induced Eryptosis			
	Septic Plasma	Healthy Plasma	*p*-Value
15 min	13.5, IQR 2.9–27.5	1.1, IQR 1.0–2.3	0.001
1 h	12.9, IQR 2.7–17.6	1.6, IQR 1.1–1.9	≤0.001
2 h	12.5, IQR 2.3–15.8	1.3, IQR 1.2–1.9	≤0.001
4 h	8.8, IQR 1.8–16.5	1.2, IQR 1.3–2.2	0.001
24 h	10.4, IQR 2.9–20	0.9, IQR 1.3–2.3	0.001

**Table 3:** Cytokines and oxidative stress level in septic patients and controls.

	Septic Patients	Controls	*p*-Value
IL-6, pg/mL	750.8, IQR 98.7–1342.8	5.4, IQR 4.4–7.8	0.0049
MPO, pg/mL	261.2, IQR 175.2–329.2	12, IQR 9.8–16.3	<0.001

## Data Availability

All data generated or analyzed during this study are included in this article. Further inquiries can be directed to the corresponding author.
